# Effects of a Brief Electronic Mindfulness-Based Intervention on Relieving Prenatal Depression and Anxiety in Hospitalized High-Risk Pregnant Women: Exploratory Pilot Study

**DOI:** 10.2196/17593

**Published:** 2020-08-11

**Authors:** Maren Goetz, Claudia Schiele, Mitho Müller, Lina M Matthies, Thomas M Deutsch, Claudio Spano, Johanna Graf, Stephan Zipfel, Armin Bauer, Sara Y Brucker, Markus Wallwiener, Stephanie Wallwiener

**Affiliations:** 1 Department of Obstetrics and Gynecology University of Heidelberg Heidelberg Germany; 2 Department of General Pediatrics, University Children's Hospital Heidelberg Germany; 3 Department of Psychology Ludwig Maximilian University Munich Germany; 4 Department of Psychosomatic Medicine and Psychotherapy University Hospital Tuebingen Tuebingen Germany; 5 Research Institute for Women's Health, Department of Women's Health University Hospital Tuebingen Tuebingen Germany; 6 Department of Women's Health University Hospital Tuebingen Tuebingen Germany

**Keywords:** pregnancy, high-risk pregnancy, hospitalization, preterm labor, anxiety, depression, psychological stress, mindfulness, stress reduction, mobile app

## Abstract

**Background:**

Peripartum depression and anxiety disorders are highly prevalent and are correlated with adverse maternal and neonatal outcomes. Antenatal care in Germany does not yet include structured screening and effective low-threshold treatment options for women facing peripartum depression and anxiety disorders. Mindfulness-based interventions (MBIs) are increasingly becoming a focus of interest for the management of such patients. Studies have shown a decrease in pregnancy-related stress and anxiety in expectant mothers following mindfulness programs.

**Objective:**

The aim of this study was to explore the clinical effectiveness of a 1-week electronic course of mindfulness on prenatal depression and anxiety in hospitalized, high-risk pregnant women. We hypothesized that participating in a 1-week electronic MBI (eMBI) could alleviate symptoms of depression and anxiety during the hospital stay.

**Methods:**

A prospective pilot study with an explorative study design was conducted from January to May 2019 in a sample of 68 women hospitalized due to high-risk pregnancies. After enrolling into the study, the participants were given access to an eMBI app on how to deal with stress, anxiety, and symptoms of depression. Psychometric parameters were assessed via electronic questionnaires comprising the Edinburgh Postnatal Depression Scale (EPDS), State-Trait Anxiety Inventory (STAI-S), and abridged version of the Pregnancy-Related Anxiety Questionnaire (PRAQ-R).

**Results:**

We observed a high prevalence of peripartum depression and anxiety among hospitalized high-risk pregnant women: 39% (26/67) of the study participants in the first assessment and 41% (16/39) of the participants in the second assessment achieved EPDS scores above the cutoff value for minor/major depression. The number of participants with anxiety levels above the cutoff value (66% [45/68] of the participants in the first assessment and 67% [26/39] of the participants in the second assessment) was significantly more than that of the participants with anxiety levels below the cutoff value, as measured with the STAI-S. After completing the 1-week electronic course on mindfulness, the participants showed a significant reduction in the mean state anxiety levels (*P*<.03). Regarding pregnancy-related anxiety, participants who completed more than 50% of the 1-week course showed lower scores in PRAQ-R in the second assessment (*P*<.05). No significant changes in the EPDS scores were found after completing the intervention.

**Conclusions:**

Peripartum anxiety and depression represent a relevant health issue in hospitalized pregnant patients. Short-term eMBIs could have the potential to reduce anxiety levels and pregnancy-related anxiety. However, we observed that compliance to eMBI seems to be related to lower symptoms of pregnancy-related stress among high-risk patients. eMBIs represent accessible mental health resources at reduced costs and can be adapted for hospitalized patients during pregnancy.

## Introduction

Mental disorders are highly prevalent during pregnancy and impose a major burden for the expectant mother, her environment, and the health care system. The prevalence rate of depression has been reported to be 11%-17% in women during the peripartum period, depending on the gestational age [1,2]. For instance, using validated screening instruments, Bennett et al found that depression was prevalent in 11.1% of the women in their third trimester [1]. Apart from depression, many pregnant women show symptoms of pregnancy-related stress and anxiety. Recently, the prevalence rate of antenatal and postnatal anxiety disorders was reported to be approximately 15% [3-5]. The reasons for developing a mental disorder during the peripartum period are still not sufficiently understood. However, it is clear that pregnancy and the puerperium period are times of particular vulnerability and emotional distress. Popular interpretations of what pregnancy should be like, for example, that pregnancy is a happy time when women enjoy the satisfaction of fulfilling a valuable reproductive role in the society, negatively affects those who are already vulnerable to distress and low moods [6,7].

Hormonal changes in pregnant women may play a major role in their emotional well-being, and associations of these hormonal changes with prolactin, steroids, and cortisol levels have been discussed previously [8,9]. If complications such as preterm labor develop during pregnancy, it can be assumed that maternal emotional distress will increase further. Worries about the course of pregnancy and the child’s health are common burdens and contribute to negative psychological reactions such as anxiety or emotional lability [10,11]. Hence, it is not surprising that available research on women hospitalized with high-risk pregnancies reports rates of anxiety and depression of up to 40% [12-14].

Peripartum mental disorders have been identified as a potential risk factor for adverse obstetric, fetal, and neonatal outcomes. Studies suggest that there is a link between untreated symptoms of depression, anxiety, or stress and increased rates of birth complications, preterm birth, and fetal or infant growth impairment [15,16]. Considering the high prevalence of mental disorders and their adverse consequences, it is all the more surprising that antenatal care does not yet include structured screening and effective treatment options for women facing this problem. In Germany, the mental health state of pregnant women so far has only been taken into account in regular care by an entry in the maternity record labeled as “mental distress.” However, this is based on a subjective assessment of the attending gynecologist and is empirically not proven. Indeed, hospitalized pregnant women are not routinely screened or offered psychological support either. Thus, mental disorders during pregnancy are overlooked in up to 80% of cases, and only 20% of those affected receive appropriate treatment [17]. In addition, affected women are difficult to reach, even with a correct diagnosis, and adequate treatment of peripartum mental disorders is particularly challenging, as drug therapy is often rejected for fear of harming the fetus [18].

Interest in mindfulness-based programs has increased substantially during the last 2 decades [19]. Several potential mechanisms underlying the efficacy of mindfulness-based interventions (MBIs) have been proposed, including exploring internal experiences such as cognitions, emotions, and sensations, affect regulation, decision-making, self-management, and relaxation [20,21]. Recent studies on MBI for pregnant women have shown generally positive effects, including decline in the symptoms of depression, anxiety, pregnancy-related stress, and increased childbirth self-efficacy [22-25].

Although the benefits of MBIs are well supported, less attention has been paid to the potential harm of MBIs. Recent studies and popular media articles suggest that mindfulness or meditation practices might have negative effects such as increased anxiety and unpleasant experiences [26,27]. However, based on the scientific literature, this aspect of MBI is not yet sufficiently understood.

Incorporating mindfulness programs in the prenatal care structure could offer vulnerable pregnant women a stigma-free strategy for addressing these issues [24]. The stigma attached to mental illness represents the major barrier to disclosure and to seeking help in the perinatal period [28]. A recent study of Moore et al showed that many women in the peripartum period were concerned about feeling like or being seen as a “bad mother” if they had a mental illness. They also feared that disclosing symptoms to a health care provider would lead to external stigma. Electronic programs could improve women’s disclosure and strengthen treatment uptake and compliance [29].

The general conclusions about the efficacy of electronic MBI (eMBI) programs cannot be drawn according to the current data on mindfulness-based stress reduction during pregnancy. Most of the related research to date lacks methodologically rigorous trials with a randomized-controlled approach [30-32]. A review evaluating the effectiveness of MBI in the perinatal period found that studies tended to focus on healthy rather than the clinical populations [31]. So far, only few studies have included psychiatrically high-risk pregnant women. The results showed a significant reduction in depression or anxiety scores after the intervention, while mindfulness skills increased. Moreover, the interventions appeared to have a long-term effect on the maternal-fetal attachment domain, which was measured via the maternal-fetal attachment scale [10,33].

Pregnant women engage regularly with digital health technology and they were found to be willing to participate in web-supported perinatal interventions [34]. Indeed, web-based or mobile interventions may represent a promising approach particularly for pregnant women with preterm labor whose mobility is often limited [35]. Nevertheless, studies involving electronic mindfulness programs are sparse. While several meta-analyses have assessed the effectiveness of face-to-face MBIs, evidence supporting the applicability and effectiveness of MBIs when delivered through web-based or mobile devices is clearly lacking [36,37]. A review and meta-analysis of randomized controlled trials (RCTs) estimating the overall effects of electronic MBIs on mental health showed a small but significant beneficial effects on depression, anxiety, well-being, and mindfulness. However, the effect sizes were not significantly related to the study quality [38].

The aim of our study was to investigate the effectiveness of a brief electronic 1-week course of mindfulness on prenatal depression and anxiety in a setting of hospitalized patients with high-risk pregnancies. We hypothesized that attending a 1-week eMBI course can alleviate the symptoms of depression and anxiety during the hospital stay.

## Methods

### Recruitment of the Participants and Study Design

**Figure 1:**
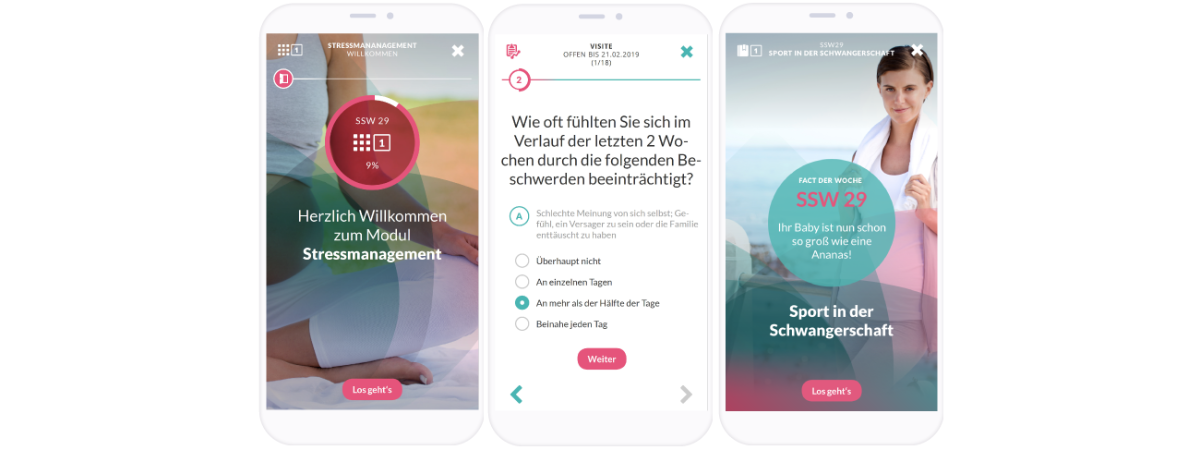
Screenshots of the electronic mindfulness-based intervention app. The first screen shows the home screen, the second screen shows the electronic assessment of the patient reported outcomes, and the third screen shows the digital pregnancy counselor.

This pilot study was conducted at the University Women’s Hospital in Heidelberg, a perinatal center of the highest level, performing over 2300 deliveries per year. Pregnant women hospitalized due to high-risk pregnancies were asked to participate in the study. The criteria for eligibility included age of 18 years or older, fluency in the German language, a gestational age of ≥24 and ≤34 weeks, and the ability to access to the internet. Women were not eligible to participate if they were expecting multiples. In total, 90 inpatients were asked to participate, of whom 68 women agreed. The reasons for not participating included lack of interest, scheduling conflicts (clash of dates due to lack of time), or severe pregnancy complications. After enrolling in the study, every participant was provided with a tablet and free wireless internet service and access to the eMBI course (Figure 1). The app was designed and developed by an interdisciplinary team of gynecologists, psychologists, and midwives by the Institute for Women's Health Tuebingen, Germany. The study participants took part in a brief 1-week course of mindfulness through an eMBI based on the *mindmom* app, which is currently being examined in a prospective randomized trial (trial registration DRKS 00017210). The RCT aims to examine the clinical effectiveness and cost-effectiveness of an eMBI in a sample of pregnant women during the third trimester of pregnancy who were screened positive for emotional distress according to the Edinburgh Postnatal Depression Scale (EPDS). The screening is administered while attending ambulatory prenatal care either with a registered gynecologist or at one of the study centers. The participants were randomized in 1:1 into the intervention (eMBI) or control (usual treatment) group.

The original intervention was used prenatally in an established concept of 8 weekly 45-min sessions [39]. In this pilot study, the time frame was tightened to a 1-week version, including a total of three 45-min modules on mindfulness (Table 1). The pilot study was carried out to optimize the user-friendliness of the mobile app by making appropriate adjustments. In addition to the questionnaires, semistructured interviews were conducted. Feedback from the participants on user-friendliness and suggestions for improvement (semistructured, qualitative evaluation) were taken into account in the final concept of the content and structure of the mobile app. We chose to use an inpatient population as the convenience sample for a population at heightened risk for mental health conditions and to explore whether eMBIs could represent accessible mental health resources to support hospitalized patients during pregnancy.

**Table 1:** Overview of the mindmom app content.

Contents	Module 1 (day 1)	Module 2 (day 3)	Module 3 (day 5)
Topic	Fears and worries about birth and parenting	Coping with stress	Me and my baby
Psychoeducation	Occurrence of pregnancy-related stress, emergence of mental vicious circles, individual sources of strength	Stress and the effects on the body (eg, during pregnancy, birth), favorable conditions for uncomplicated birth process	Positive effects of self-care, caring contact with the baby during pregnancy
Skills	Exit from the vicious circle through your own sources of strength, feel-good place	Encouraging sentences, reward cards	Contact with the baby/positive attitude toward the child, benevolent companions
Mindfulness	Mindful breathing	Mindful body scan	Mindful “loving kindness”

Through mediation of psychoeducational content and cognitive behavioral therapy-related approaches, the app teaches participants how to deal with stress, pregnancy-related anxiety, and symptoms of depression. Thus, it promotes the autonomy of the mother-to-be regarding the upcoming birth and during the initial days and weeks with the newborn. The app contains instructional videos and audio files, interactive worksheets, and a personal “skills box” to collect exercises, videos, and texts, which the participants found helpful. After the 1-week course, participants had the opportunity to continue accessing the exercises. All the participants received scientifically validated information about pregnancy and birth via a pregnancy counselor. The main topics were the physical changes in pregnancy, the birth process, pain relief during birth, bonding between parents and child, breastfeeding, and tips on the formalities related to birth. No financial compensation was offered to the participants. All questionnaires were completed digitally prior to and after the 1-week course. In addition, we gathered medical and sociodemographic data that were double-checked against the hospital records (Table 2). Data were collected via the mindmom app based on electronic patient reported outcomes.

To assess the psychometric data on depression and symptoms of anxiety, the following instruments were used: EPDS, State-Trait Anxiety Inventory (STAI), and Pregnancy-Related Anxiety Questionnaire (PRAQ).

**Table 2:** Questionnaire assessment.

Visiting schedule, data captured			Questionnaires to be filled
**Visit 1 (day 1)**			
	Sociodemographic data (age, education level, marital status, income, occupation, number of children)	Self-designed^a^	
	Medical history (gravidity, parity, fertility treatment)	Self-designed	
	Psychiatric history	Self-designed	
	Use of the internet	Self-designed	
	Symptoms of depression and pregnancy-related anxiety	EPDS^b^, STAI^c^	
	Fear of childbirth	PRAQ-R^d^	
**Visit 2 (day 7)**			
	Symptoms of depression and pregnancy-related anxiety	EPDS, STAI	
	Fear of childbirth	PRAQ-R	
	Feasibility and acceptance of the mindmom app	Self-designed	

^a^The questionnaires were not validated as they were self-designed.

^b^EPDS: Edinburgh Postnatal Depression Scale (validated questionnaire).

^c^STAI: State-Trait Anxiety Inventory (validated questionnaire).

^d^PRAQ-R: Pregnancy-Related Anxiety Questionnaire abridged version (validated questionnaire).

#### EPDS

The EPDS is a 10-item self-rating scale that assesses the depressive symptoms during the peripartum period. It was originally developed by Cox et al in 1987 [40] and translated into German by Bergant et al [41]. The EPDS is used for research purposes and has been proven to be an efficient and effective way of identifying patients at risk for perinatal depression. With a cutoff value of 9 (EPDS>9), the sensitivity of detecting a clinically significant depression is 0.96, the specificity is 1.00, and the positive predictive value is 1.00 [40-42]. The scale reached a good to excellent internal consistency in our sample (Cronbach α=.91 in the first assessment and α=.81 in the second assessment).

#### STAI

Symptoms of anxiety are assessed with the STAI. The STAI was developed by Spielberger et al [43] and translated into German by Laux et al [44]. Based on Cattell’s theory of anxiety, the STAI consists of 2 scales (STAI-S [STAI-State] and STAI-T [STAI-Trait]), each comprising 20 items to separately assess anxiety as a general trait or as a temporary condition. The 2 scales can be used together or separately. The items are answered on the basis of a 4-point Likert scale; the answers (1-4) are added to the total value, with negative polarized items being reversed. A total value of 20 implies “absolute absence of anxiety” and a maximum score of 80 means “highest level of anxiety” [45]. The STAI was validated for pregnancy by Grant et al [46]. The best cutoff of the STAI for perinatal samples was found to be 40, which matches the original cutoff [43]. The scale reached a good to excellent internal consistency in our sample (STAI-S: Cronbach α=.95 at first and α=.93 at second assessment; STAI-T: Cronbach α=.89 at first and α=.91 at second assessment).

#### PRAQ

Pregnancy-related anxiety is assessed with the PRAQ. The questionnaire was originally developed by van den Bergh [47] and abridged by Huizink et al (PRAQ-R). It consists of 10 items. The response categories range from “never applicable” to “very strong/very often true” on a 5-point Likert scale. The items are added to a total sum score (maximum score 10), with a higher sum score indicating a higher level of pregnancy-related anxiety. In our sample, the PRAQ-R showed an acceptable to good internal consistency with a Cronbach α of .84 at the first assessment and α of .76 at the second assessment for the whole instrument.

### Statistical Analyses

All analyses were conducted using the SPSS software (IBM Corp, v. 24.0.0.0). Since the distributions of the self-report scales did not deviate from the normal distribution (*P*>.18 in Kolmogorov-Smirnov-test), we chose a parametric analysis strategy for the parametric variables. Due to scale-specific amounts of the missing values, the number of valid cases varies between the analyses. We considered sociodemographic data (eg, age), medical data (eg, gestational age), and the self-report data (eg, EPDS) for this procedure. The missing completely at random test results were not significant (*χ*²_341_=334.4, *P*=.59), indicating that missing values were at random and that subpopulations were representative for the total sample [48].

First, the descriptive statistics of the sample characteristics in the relevant sociodemographic, medical, and self-reported variables were reported according to the levels measured. Second, the frequency of the cases scoring above the EPDS cutoff (>9) were compared to the expected frequency (11.1%) according to the review of Bennet et al [1] by using the chi-square test. The same analysis was used to determine the frequency of the women scoring above and below the STAI cutoff (>40) for equality. Third, the EPDS, STAI, and PRAQ-R scores were tested for a decline between the first and second measurements by using two-tailed *t* tests for paired samples. Finally, high versus low treatment compliance was tested for differences regarding the symptom levels by using *t* tests for independent means. The two-sided critical α-error was set to α=.05. Due to the exploratory nature of the analyses, the α errors were not Bonferroni-adjusted. To estimate the effect sizes, we computed ω² = *χ^2^*/N for chi-square tests and ω² for *t* tests. ω² is a population-based estimator of the explained variance. ω²=0.01 or ω²=0.01 is interpreted as small effects, ω²=0.09 or ω²=0.06 as medium-sized effects, and ω²=0.25 or ω²=0.14 as large effects [49].

## Results

### Sociodemographic Data, Medical Data, and Self-Reports

In total, 68 hospitalized pregnant women were included in the study, 39 of whom completed the full 1-week electronic mindfulness course. The most common diagnoses of the participants are listed in Table 3. Of the 68 participants, the information for 1 participant regarding the diagnosis for hospitalization was missing; therefore, the information of 67 participants is provided in Table 3.

A total of 29 participants who completed the baseline visit were lost to follow-up. Thus, the overall completion rate was 57% (39/68). The nonparametric sociodemographic characteristics of the study population are summarized in Table 4. The parametric demographic, medical, and self-report data are summarized in Table 5.

**Table 3:** Cases diagnosed at admission for hospitalization (N=67, multiple answers possible).

Diagnosis at admission	Valid cases, n (%)
Cervical insufficiency	27 (40)
PROM^a^/premature labor	19 (28)
IUGR^b^/oligohydramnios	10 (15)
Vaginal bleeding	8 (12)
Infections	5 (7)
Placental disorder	4 (6)
Fetal malformation	4 (6)
Polyhydramnios	3 (4)
Gestational diabetes	3 (4)
Other	9 (13)

^a^PROM: prelabor rupture of membranes.

^b^IUGR: intrauterine growth restriction.

**Table 4:** Analysis of the nonparametric sample characteristics.^a^

Nonparametric data		Valid cases, n (%)
**Civil status**		
	Married and living together	49 (73)
	Married and living apart	1 (1)
	Single	17 (25)
**Number of children**		
	0	40 (59)
	1	23 (34)
	≥2	5 (7)
**Education**		
	Lower secondary qualification	5 (7)
	Higher secondary qualification	22 (32)
	University entrance qualification	41 (60)
**Occupation**		
	Unemployed	33 (48)
	Part-time	9 (13)
	Full-time	26 (38)
**Income (1 €=1.15 USD)**		
	<1500 €	18 (27)
	1500-4999 €	39 (59)
	>5000 €	9 (14)
**Gravidity**		
	1	30 (44)
	2	27 (40)
	≥3	11 (16)
**Parity**		
	0	39 (58)
	1	23 (34)
	≥2	5 (7)
**Outpatient** **psychiatric psychotherapeutic treatment**		
	Never	53 (78)
	Earlier	14 (21)
	Current	1 (1)
**Inpatient psychiatric/psychotherapeutic treatment**		
	Never	60 (91)
	Earlier	5 (8)
	Current	1 (2)
**Current mental illness**		
	No	64 (97)
	Yes	2 (3)
**Mental illness in family**		
	No	50 (75)
	Yes	17 (25)
**Former medication for states of depression or anxiety^b^**		
	No	57 (84)
	Yes	11 (16)
**Current or past prepartum anxiety disorder**		
	No	62 (91)
	Yes	6 (9)
**Current** **or past postpartum anxiety disorder**		
	No	66 (99)
	Yes	1 (1)
**Current or past postpartum depression^b^**		
	No	66 (98)
	Yes	1 (1)

^a^The total valid number of cases varied between 66 and 68.

^b^There were no cases with current or past prepartum depression or of those on medications.

**Table 5:** Analysis of the parametric sample characteristics.

Parametric data	Sample size (n)	Range	Mean (SD)	Standard error
Maternal age (years)	68	22-41	32.07 (4.74)	0.58
Weight before pregnancy (kg)	68	30-138	70.32 (19.77)	2.40
Body height (cm)	68	150-183	165.19 (6.93)	0.84
Current weight (kg)	68	42-146	79.87 (19.11)	2.32
Gestation age (weeks)	63	24-34	30.17 (3.17)	0.40
EPDS^a^ score (T1)^b^	67	0-25	8.39 (5.59)	0.68
EPDS score (T2)^c^	39	3-20	8.62 (4.13)	0.66
STAI-S^d^ score (T1)	68	28-79	46.66 (11.54)	1.40
STAI-S score (T2)	39	29-65	43.81 (10.09)	1.62
STAI-T^e^ score (T1)	68	22-53	38.18 (8.01)	0.97
STAI-T score (T2)	39	24-54	38.43 (8.46)	1.36
PRAQ-R^f^ score (T1)	68	10-40	22.83 (7.31)	0.89
PRAQ-R score (T2)	39	10-39	20.69 (6.09)	0.98

^a^EPDS: Edinburgh Postnatal Depression Scale.

^b^T1: first assessment.

^c^T2: second assessment.

^d^STAI-S: State-Trait Anxiety Inventory (State scale).

^e^STAI-T: State-Trait Anxiety Inventory (Trait scale).

^f^PRAQ-R: Pregnancy-Related Anxiety Questionnaire abridged version.

### Tests on the Distribution of the Cases Below and Above Cutoffs

Table 6 shows the observed and expected case numbers for the EPDS, the STAI-S, and the STAI-T cutoffs at both assessments. The chi-square tests were highly significant for the EPDS at both assessments (*P*<.001) with large empirical effects (ω²=0.778 at first and ω²=0.908 at second assessment). With 39% (26/67) of the participants at the first assessment and 41% (16/39) of the participants at the second assessment scoring above the cutoff (>9), there were significantly more participants with depressive symptoms in the sample than expected (11.1%). The chi-square tests were also significant for the STAI-S at both assessments (*P*=.008 at first and *P*=.04 at second assessment) with medium-sized empirical effects (ω²=0.105 at first and ω²=0.111 at second assessment). With 66% (45/68) of the participants at the first assessment and 67% (26/39) of the participants at the second assessment scoring above the cutoff (>40), there were significantly more state anxious participants in the sample than expected (34/68; 50%). The chi-square tests were not significant for the STAI-T at either assessment (*P*=.22 at first and *P*=.63 at second assessment). With 42.6% (29/68) of the participants at the first assessment and 46.2% (18/39) of the participants at the second assessment scoring above the cutoff (>40), the number of trait anxious participants was not significantly higher in the sample than expected (34/68; 50%). The power to find large effects in this analysis was sufficient for large effects (ω²=0.25, 1–β=.98 at the first and 1–β=.87 at the second assessment). However, medium-sized (ω²=0.01, 1–β=.70 at the first and 1–β=.47 at the second assessment) and small effects (ω²=0.01, 1–β=.13 at the first and 1–β=.10 at the second assessment) cannot sufficiently be ruled out.

**Table 6:** Chi-square tests on the distribution of the cases below and above the cutoffs.

Assessments, cutoffs		Observed cases (n)	Expected cases (n)	Chi-square (*df*)	*P* value
**EPDS^a^** **(T1)^b^**				52.1 (1)	<.001
	≤9	41	60		
	>9	26	7		
**EPDS (T2)^c^**				35.4 (1)	<.001
	≤9	23	35		
	>9	16	4		
**STAI-S^d^** **(T1)**				7.1 (1)	.008
	<40	23	34		
	≥40	45	34		
**STAI-S (T2)**				4.3 (1)	.04
	<40	13	20		
	≥40	26	20		
**STAI-T^e^** **(T1)**				1.5 (1)	.22
	<40	39	34		
	≥40	29	34		
**STAI-T (T2)**				0.2 (1)	.63
	<40	21	19.5		
	≥40	18	19.5		

^a^EPDS: Edinburgh Postnatal Depression Scale.

^b^T1: first assessment.

^c^T2: second assessment.

^d^STAI-S: State-Trait Anxiety Inventory (State scale).

^e^STAI-T: State-Trait Anxiety Inventory (Trait scale).

### Tests on the Changes Between the First and Second Assessments

Table 7 summarizes the descriptive and inferential results of the paired sample *t* tests on the changes between the first and the second assessment in the subsample of 39 participants who were eligible at both assessments. No significant change was found in the EPDS, STAI-T, or PRAQ-R scores (*P*>.20). The power to detect large (ω²=0.14, 1–β>.99) and medium-sized effects (ω²=0.06, 1–β=0.86) was sufficient for these analyses. At the same time, with a power of 1–β=0.23, small effects (ω²=0.01) cannot be ruled out. However, the STAI-S scores significantly declined for these patients between the first and the second assessments (*P*=.03) with a small effect of ω²=0.051 (ie, 5.1% of the variance of the changes between the first and the second assessment can be explained by the passing of time).

**Table 7:** Results of the paired sample t tests on the changes between the first and second assessments (n=39).

Assessments		Mean (SD)	Standard error	*t (df)*	*P* value
**EPDS^a^** **score**				–0.370 (38)	.71
	T1^b^	8.41 (4.77)	0.76		
	T2^c^	8.62 (4.13)	0.66		
**STAI-S^d^** **score**				2.277 (38)	.03
	T1	46.65 (11.35)	1.82		
	T2	43.81 (10.09)	1.62		
**STAI-T^e^** **score**				0.325 (38)	.75
	T1	38.60 (7.39)	1.18		
	T2	38.43 (8.46)	1.36		
**PRAQ-R^f^** **score**				1.317 (38)	.20
	T1	21.63 (6.08)	0.97		
	T2	20.69 (6.09)	0.98		

^a^EPDS: Edinburgh Postnatal Depression Scale.

^b^T1: first assessment.

^c^T2: second assessment.

^d^STAI-S: State-Trait Anxiety Inventory (State scale).

^e^STAI-T: State-Trait Anxiety Inventory (Trait scale).

^f^PRAQ-R: Pregnancy-Related Anxiety Questionnaire abridged version.

### Differences Between High and Low App Engagement Regarding Symptom Levels

To examine the differences between the high and low app engagement regarding the symptom levels at the second assessment, we defined a group of participants that completed more than 50% of the 1-week course modules. Furthermore, we dummy coded noncompliant as “0” and compliant as “1” for >50% of all the 3 modules. There was no significant difference between high (8.96 [SD 4.30]) and low app engagement (8.30 [SD 4.05]) regarding EPDS scores at the second assessment (*t_37_*=–0.49, *P*=.62). Moreover, there were no significant differences regarding the STAI-S scores (*t_37_*=–0.60, *P*=.55) or the STAI-T scores (*t_37_*=–0.60, *P*=.55) at the second assessment between high (STAI-S: 44.82 [SD 10.56]; STAI-T: 39.26 [SD 8.81]) and low app engagement (STAI-S: 42.85 [SD 9.79]; STAI-T: 37.63 [SD 8.27]). The power to find large effects in these analyses was insufficient for large (ω²=0.14, 1–β=.68), medium-sized (ω²=0.06, 1–β=.33), and small effects (ω²=0.01, 1–β=.09). Consequently, the effects of any size cannot sufficiently be ruled out.

However, a significant, medium-sized difference regarding PRAQ-R scores was observed at the second assessment (*t_37_*=2.03, *P*<.05). If participants completed more than 50% of all the modules, they had significantly lower PRAQ-R scores (18.74 [SD 4.49]) than those with low app engagement (22.54 [SD 6.90]) at the second assessment. Overall, 7.4% (ω²=0.074) of the PRAQ-R score variance at the second assessment can be explained by the group variable “high vs low app engagement.”

## Discussion

### Principal Results

In our study, the prevalence of depression and anxiety among hospitalized high-risk pregnant women was found to be high. At the baseline assessment, 39% (26/67) of the study participants achieved EPDS scores above the cutoff value for a minor/major depression and 66% (45/68) of the women had high levels of anxiety as measured with the STAI-S. Therefore*,* our results reinforce the findings of previous studies on depression and anxiety disorders in perinatal populations demonstrating regularly higher EPDS and STAI scores than the expected ranges in nonpregnant populations [50]. In our study, we used a meta-analysis including 19,284 patients for comparison that reported EPDS scores ≥10 in 11.7% and 11.1% of the women in the second and third trimester, respectively [1]. Regarding anxiety symptoms during pregnancy, recent reviews found pooled prevalence rates of 15%-23% across trimesters [5,51]. The study selection of these reviews was restricted to samples of pregnant women recruited through general obstetric/prenatal units. By contrast, our sample included hospitalized high-risk pregnant patients, in whom prevalence rates are considerably higher.

When compared to recently published studies with inpatient samples, we found similar tendencies for anxiety and depression levels during pregnancy. Regarding antenatal depression assessed via the EPDS, a study in Singapore found that the rate of major depression in a sample of high-risk pregnancies (11%) was higher than that in an unspecified obstetric risk cohort (4.3%) [14]. Dagklis et al presented similar results with high depression rates of 24.3% and 28% in 2 different high-risk pregnancy unit samples [52,53]. The scores in our sample exceed the aforementioned study results considerably, with up to 41% (16/39) of the participants reaching or exceeding the EPDS cutoff value. In a recent study examining depression, anxiety, and attachment among women hospitalized in an antepartum unit, screening identified over one-third (36%) of the participants to be at risk for depression (EPDS score ≥10) and almost half (47%, 46/98) reported elevated state anxiety (STAI-S ≥40) [54]. Likewise, Barber and Starkey showed that hospitalized pregnant women had significantly higher state anxiety levels, with 47% of the women showing STAI-S scores above the cutoff compared to normative data from nonpregnant samples [55]. These results are in line with our sample, with STAI-S scores >40 for 66% (45/68) of the participants.

In direct comparison, it becomes clear that hospitalized pregnant women represent a vulnerable group, showing rates of anxiety and depression more than three times greater than those reported in nonclinical samples. Despite the high rates of anxiety and depressive symptoms, a surprisingly low number of participants were receiving mental health treatment. In inpatient obstetric settings, where access to individual psychotherapy is often extremely limited, eMBIs could provide an easily accessible support option. Incorporating low-threshold eMBIs not only in hospital care but also in outpatient practices could minimize the stigma of starting mental health treatment.

### Measurement Tools

For our sample, we decided to choose a cutoff value of >40 for STAI and a cutoff value >9 for EPDS, which is in line with that reported in recent studies on the use of EPDS and STAI as valid screening tools for depression and anxiety during pregnancy. Tendais et al found optimal cutoffs for STAI-S as 40 and EPDS as 9 during pregnancy [56]. Regarding the accuracy of the EPDS in identifying depression and other mental disorders, Howard et al reported a likelihood ratio of 9.8 for the EPDS [3]. The fact that the EPDS also performs well in screening for depression and anxiety in high-risk pregnant women was confirmed by Thiagayson et al, who recommended further psychiatric assessments for women with a score ≥9, as found in our study [14]. For the STAI, Barnett and Parker recommended cutoffs of high (≥40), moderate (32-33), or low (≤25) anxiety on the basis of the mean trait scores of a sample of 94 primiparae [57]. Grant et al found that a cutoff >40 for both state and trait scales yielded optimal sensitivity (80.95%), specificity (79.75%), and positive predictive value (51.5%) to determine cases of anxiety in the third trimester of pregnancy [46]. In our sample, there were 59% (40/68) primiparae, and the mean gestational age was 30.14 weeks; thus, the values compare well. In all, the EPDS and STAI prove to be effective screening tools that are frequently used in studies including high-risk pregnant women.

### Effectiveness of eMBI in Reducing Anxiety

The latest reviews about eMBI during pregnancy suggest that web-based interventions targeted at improving mental health may be beneficial during the peripartum period. However, the findings and their generalizability are limited both by the heterogeneity of the interventions and study designs and by methodological limitations. Pooled results of non-RCTs reporting outcomes on anxiety, depression, and perceived stress showed a significant benefit for the mindfulness group, but this review found no differences between the mindfulness intervention and control groups in RCTs [58]. Another review showed significant reductions in depression, anxiety, and stress by means of preanalyses and postanalyses, each with small to medium effect sizes [31]. However, between-group analyses failed to find any significant postintervention benefits of MBIs in comparison to control conditions.

In our pilot study without a comparison group, a significant reduction in the mean state anxiety levels was found after completing the 1-week eMBI (*P*<.03). Recent studies including other MBIs during pregnancy found similar effects with significantly lower STAI scores (*P*<.001) after interventions such as yoga, music therapy, or progressive muscle relaxation [50,59]. Compassion-focused therapy represents another promising approach for effectively treating depression and anxiety in perinatal populations [60]. The equivalence of compassionate mind training on the constructs of self-confidence, inadequate self-criticism, and self-compassion and the superior performance of compassionate mind training in reducing depression and anxiety scores in the latest studies suggest that compassion-focused therapy offers additional benefits beyond the current gold standard of cognitive behavioral therapy [61,62].

### App-Related Patient Engagement

As one of our key results, we found a small, yet significant reduction in the mean state anxiety levels after completing the 1-week electronic course of mindfulness (*P*<.03). Concerning engagement with the app, participants who completed more than 50% of all the 3 modules showed lower scores for the PRAQ-R at the second assessment (*P*<.05). Our sample population showed an overall completion rate of 57% (39/68). We decided to consider an individual completion rate of more than 50% of the overall module in at least 2 of 3 course modules as compliant, as similar cutoffs have been used before. For instance, previous studies on mindfulness-based programs have considered participants who completed at least 50% of sessions as having fulfilled an adequate minimum amount of the course [30]. Hence, our compliance rate is comparable to that reported in similar studies, which reported even lower compliance rates from 21% to 35% [30,63]. Regarding the nature of this very specific study population, the majority of the dropouts can be explained by hospital discharge before the second assessment. Women who are no longer considered to be at high risk of preterm delivery might not see any reason to continue the eMBI, as they have already experienced symptom relief due to the end of hospitalization. Nonetheless, the overall dropout rate of the course was high (29/68, 43%). The reasons for dropout included discharge from the hospital, actually giving birth prematurely, severe stress and concerns, or adverse pregnancy complications. Studies have shown that although face-to-face mindfulness courses for pregnancy have demonstrated good levels of adherence to the course with completion rates of over 85% [39], adherence after online courses tended to be more difficult. The reasons for low levels of motivation might include rare contact with the treatment team/researchers or a lack of social support from the other participants [30].

### Limitations

As our pilot study design is not an RCT, the changes in the mean state anxiety levels after the 1-week course of mindfulness cannot be clearly attributed to the eMBI. Urech et al [35] assessed the efficacy of a web-based cognitive behavioral stress management training in women with preterm labor and found no significant differences in the psychological parameters between the intervention and the control group (based on distraction). They hypothesized that the psychological well-being improves automatically during the course of pregnancy since participants in both groups showed lower stress-related psychological levels [35]. However, another study suggested that differences in the mean change of the STAI scores between the intervention and control groups were not solely due to feelings and symptoms changing as gestation progresses [50]. Considering the length of the eMBI, most studies report an average duration of 6-8 weeks of intervention [23,39]. In comparison, our 1-week electronic mindfulness course may seem rather short. However, a course duration of 8 weeks would be unsuitable for inpatients. Newham et al already proved that even a single session of yoga reduced both the subjective and physiological measures of state anxiety (STAI-S and cortisol), and this class-induced reduction in anxiety remained at the final session of the intervention [64]. Likewise, studies examining the immediate effects of an intervention reported significantly lowered STAI scores after a single session of complementary therapy-based interventions. The mean post-intervention scores were consistently significantly lower than the baseline intervention scores after all single-session interventions [50]. These facts underscore our results and demonstrate that short-term interventions can be beneficial, especially for those who have the greatest need. However, the small sample size limits the statistical power to detect the effects as well as the generalizability. The large number of statistical tests used in this study increases the global α error; thus, random effects cannot be ruled out sufficiently.

### Conclusions

Peripartum anxiety and depression are highly relevant health issues in hospitalized pregnant patients. Despite high prevalence rates and the patients’ need for regular mental health support, only a very small proportion receives adequate treatment. In this pilot study, we could show that our 1-week course of mindfulness may be an effective brief intervention that could have the potential to positively influence childbirth anxiety and pregnancy-related stress among high-risk patients. eMBIs may provide an easily accessible and effective means to support pregnant women in managing anxiety and stress during their inpatient stay and beyond. RCTs with confirmatory analyses in larger samples are needed to confirm the effectiveness of mindfulness in promoting perinatal mental health and to study the potential for harm in mindfulness-based programs.

## Abbreviations

eMBI: electronic mindfulness-based intervention
EPDS: Edinburgh Postnatal Depression Scale
MBI: mindfulness-based intervention
RCT: randomized controlled trial
PRAQ-R: Pregnancy-Related Anxiety Questionnaire abridged version
STAI-S: State-Trait Anxiety Inventory (State scale)
STAI-T: State-Trait Anxiety Inventory (Trait scale)
